# Relationship between psychosocial stress-induced prefrontal cortex activity and gut microbiota in healthy Participants—A functional near-infrared spectroscopy study

**DOI:** 10.1016/j.ynstr.2022.100479

**Published:** 2022-08-12

**Authors:** Kao Yamaoka, Nobuo Uotsu, Eiichi Hoshino

**Affiliations:** a FANCL Corporation Research Institute, 12-13 Kamishinano, Totsuka-ku, Yokohama, Kanagawa, 244-0806, Japan; b Keio University Global Research Institute (KGRI), 2-15-45 Mita, Minato-ku, Tokyo, 108-8345, Japan

**Keywords:** Psychosocial stress, Gut microbiome, Prefrontal cortex, Depression, Functional near-infrared spectroscopy, ANOVA, Analysis of variance, BD, Bipolar disorder, BMI, Body mass index, bpm, Beat per minute, CH, Channel, CRH, Corticotropin-releasing hormone, deoxy-Hb, Deoxygenated hemoglobin, dlPFC, Dorsolateral prefrontal cortex, DNA, Deoxynucleic acid, fMRI, Functional magnetic resonance imaging, fNIRS, Functional near-infrared spectroscopy, FP, Frontal pole, GABA, Gamma Amino Butyric Acid, HPA-axis, Hypothalamic-pituitary-adrenal axis, IFG, Inferior prefrontal gyrus, MDD, Major depressive disorder, MIST, Montreal Imaging Stress Task, oxy-Hb, Oxygenated hemoglobin, PANAS, Positive and Negative Affect Schedule, PET, Positron emission tomography, PFC, Prefrontal cortex, PMC/SMA, Pre-motor cortex/supplementary motor area, POMS2, Profile of Mood States 2 short version, SSES, State Self-Esteem Scale, STAI, State-Trait Anxiety Inventory, VAS, Visual analog scale

## Abstract

Brain and gut microbes communicate in a bidirectional manner with each affecting a person's response to psychosocial stress. Although human studies demonstrated that the intake of probiotics can alter stress-related behavior in both patients and healthy participants, the association between stress-related brain functions and the gut microbiota has mostly been investigated in patients with depression. However, the response to psychosocial stress differs, even among healthy individuals, and elucidating the natural state of the gut microbiota would broaden the understanding of responses to psychosocial stress. We investigated the relationship between psychosocial stress response in the prefrontal cortex and the abundance of gut microbes in healthy male participants. The participants were exposed to psychosocial stress during a task while brain activation data were recorded using functional near-infrared spectroscopy. The heart rate and subjective stress were recorded, and fecal samples were collected. The stressful condition was accompanied by high subjective stress, high heart rate, and higher prefrontal activation in the right pre-motor cortex/supplementary motor area, right dorsolateral prefrontal cortex, right frontal pole, and right inferior prefrontal gyrus. The psychosocial stress response in the prefrontal cortex was also associated with changes in the gut microbiota abundance. The abundance of *Alistipes*, *Clostridium IV*, *Clostridium XI*, *Faecalibacterium*, and *Blautia* in healthy participants who had high psychosocial stress resembled that noted in patients with depression. These results suggest that the gut microbiota differs, among healthy participants, depending on the psychosocial stress response. We believe that this study is the first to report a direct relationship between brain function and the gut microbiota in healthy participants, and our findings would shed a new light on this field in the near future.

## Highlights

• Brain and gut microbe communication affects the response to psychosocial stress.
• Gut microbiota related to psychosocial stress in healthy individuals is unknown.
• Stress-induced brain activation was observed in the right prefrontal cortex.
• Brain activity was associated with gut microbiota related to depression.
• Gut microbiota differs depending on the response to psychosocial stress.

## 1 Introduction

Prolonged harmful stress can lead to a variety of disorders, including depression and anxiety disorders (Sapolsky, 2015; Wilson et al., 2011; Winter et al., 2018). Psychosocial stress, which involves social evaluative threat, is strongly associated with depression, which is characterized by poor social function and enhanced attention to negative social signals (Joormann and Gotlib, 2010). Responses to psychosocial stress in interpersonal and uncontrollable situations are regulated by the hypothalamic-pituitary-adrenal (HPA) axis (Kirschbaum and Hellhammer, 1994; Liu, 2017). Prolonged psychosocial stress induces a constant state of high HPA-axis activity, increased corticosteroid secretion, and sympathetic nervous system dysfunction—usually accompanied by impairments in prefrontal cortex (PFC) activity, immune system function, and behavior (Cerqueira et al., 2008). Decreased PFC function due to psychosocial stress promotes depressive and anxious behaviors and patients with depression often exhibit dysfunction in the right PFC. Thus, the right hemisphere is reported to play an important role in psychosocial stress responses (Dedovic et al., 2009; Morinaga et al., 2007; Orem et al., 2019).

### 1.1 Stress and the gut-brain axis

The microbiota-gut-brain axis is a bidirectional pathway that functions via the enteric nervous system and influences the vagus nerve, inflammatory mediators, neuroendocrine systems, and metabolite production (Cussotto et al., 2018). This system allows changes in the intestinal microbiota to influence host physiology, including brain activation and behavior. For example, the ingestion of probiotics containing *Bifidobacterium longum*, *Bifidobacterium bifidum*, *Bifidobacterium lactis*, *Lactobacillus brevis*, *Lactococcus lactis*, and *Lactobacillus casei* alters factors associated with emotional responses such as the stress response, brain activation, heart rate, and cortisol secretion (Allen et al., 2016; Bagga et al., 2018; Kanehira et al., 2011; Lew et al., 2018; Ma et al., 2021; Papalini et al., 2018; Siegel and Conklin, 2020; Takada et al., 2017; Tillisch et al., 2013). Lew et al. (2018), in their 12-week study, reported that the administration of *Lactobacillus plantarum* P8 reduced stress and anxiety in highly stressed participants. In a 4-week study on healthy female participants, Bagga et al. (2018) indicated that the administration of probiotics containing *L. casei* altered the microbiota profile and brain activation associated with emotional memory and decision-making. These results indicate that the intestinal microbiota interacts with the gut-brain axis and has an important role in functional brain activation and host behavior (Sherwin et al., 2019).

Other studies (Bailey and Coe, 1999; Bailey et al., 2010, 2011; Park et al., 2013; Tannock and Savage, 1974) have demonstrated the bidirectional nature of the gut-brain axis by indicating the brain responses influencing the gut microbial composition. These studies usually induce depression- and anxiety-like behavior or apply uncontrollable stress on animal models. Enhanced expression of corticotropin-releasing hormone (CRH) alters colonic motility, thereby changing intestinal microbiota profiles in mice with induced depressive-like behaviors (Park et al., 2013). Moreover, mice bred in stress-inducing environments have increased numbers of *Alistiles* and *Odoribacter* microbes, compared to the control mice (Bendtsen et al., 2012). These results demonstrate that the manipulation of brain functions and experimental induction of stress are accompanied by alterations in the gut microbiota of the animals. Recently, a mouse-model study showed that gut microbiota modulates brain activity and regulates stress responses and social behavior (Wu et al., 2021). However, most studies on humans have included patients with depression (Barandouzi et al., 2020; Sanada et al., 2020; Winter et al., 2018 for reviews). Compared to healthy participants, patients with depression have different abundance levels of certain microbes such as *Alistipes* (Jiang et al., 2015), *Anaerofilum* (Kelly et al., 2016), *Bifidobacterium* (Messaoudi et al., 2011), *Blautia* (Jiang et al., 2015), *Clostridium IV* (Liu et al., 2016), *Clostridium XI* (Liu et al., 2016), *Faecalibacterium*, *Lactobacillus* (Lew et al., 2018; Messaoudi et al., 2011; Takada et al., 2017), and *Odoribacter* (Liu et al., 2016). This difference may result from excessive cortisol secretion in patients with depression, which affects inflammatory cytokine production and immune activity and alters the gut microbiota (Trzeciak and Herbet, 2021). Even in healthy participants, prolonged psychosocial stress can cause psychosomatic changes similar to those in patients with depression (Cerqueira et al., 2008). Thus, healthy participants under psychosocial stress may show similar alterations in gut microbiota.

### 1.2 Current study

Several studies have examined relationship between microbiota changes and stress response by investigating the effects of probiotics on stress in healthy participants (Bagga et al., 2018; Lew et al., 2018; Siegel and Conklin, 2020); others revealed changes in the abundance of certain gut microbiota types in patients with depression (Jiang et al., 2015; Kelly et al., 2016; Messaoudi et al., 2011). However, the direct relationship between the gut microbiota under natural conditions and brain functions in response to stress have received limited attention in healthy individuals. Generalizing the relationship between stress and gut microbiota is difficult because the type (e.g., social, physical, academic) and duration of the induced stress varies between studies. Stress responses vary, even among healthy individuals (Yamaoka et al., 2021). Therefore, investigating the relationship between the stress response and gut microbiota in healthy individuals could shed light on the nature of stress in humans and elucidate important factors in preventing and/or treating stress-related disorders.

In this study, we focused on psychosocial stress, which is strongly associated with depression and anxiety disorders, and recorded the brain activity of healthy participants to examine the direct relationship between gut microbiota and brain function. To measure the physiological changes caused by psychosocial stress, we recorded the brain activity and heart rate of participants during the Montreal Imaging Stress Task (MIST) (Dedovic et al., 2005, 2009; Pruessner et al., 2005), and confirmed subjective stress at multiple timepoints. We used functional near-infrared spectroscopy (fNIRS) to measure changes in the oxygenated hemoglobin (oxy-Hb) concentration in the PFC. Compared to functional magnetic resonance imaging (fMRI), fNIRS is easier to operate, has higher temporal resolution and fewer constraints. While this is an important advantage when compared with fMRI or positron emission tomography (PET) techniques that impose additional stress (irrelevant to the task) on the participants, unlike fMRI and PET, fNIRS cannot measure changes in deep brain regions. However, as the brain activation related to psychosocial stress is mainly associated with the PFC, fNIRS was determined to be the most appropriate technique.

The main objective of this study was to investigate the relationship between psychosocial stress response in the PFC and abundance of gut microbes in healthy male participants. We hypothesized that psychosocial stress would enhance PFC activity (especially in the right hemisphere) and that this would be related to the abundance of gut microbiota associated with depression.

## 2 Materials and methods

### 2.1 Participants

We recruited and screened 194 healthy right-handed males. Only males were recruited as males tend to show higher HPA-axis stress response than females (Allen et al., 2017)—an important factor in inducing the stress response. Further, females experience hormonal changes associated with the menstrual cycle, and more likely to suffer from functional gastrointestinal disorders compared to males (Kim and Kim, 2018). Thus, by including only males, we minimized the effect of gender variances which may hinder the main outcomes. Screening included a lifestyle questionnaire, medical history check, several neuropsychological tests, questionnaires, and elementary school level mental calculation questions (Supplemental Material 1). All participants provided informed consent according to the Declaration of Helsinki guidelines. Ethical approval was obtained from the Japanese Conference of Clinical Research (UMIN000041134). Sixty individuals who met the inclusion criteria (Supplemental Material 2) participated (age range: 25–45 years; mean age ± standard deviation [SD]: 34.95 ± 6.26 years).

### 2.2 Behavioral neuroimaging task and data analysis

**Fig. 1:**
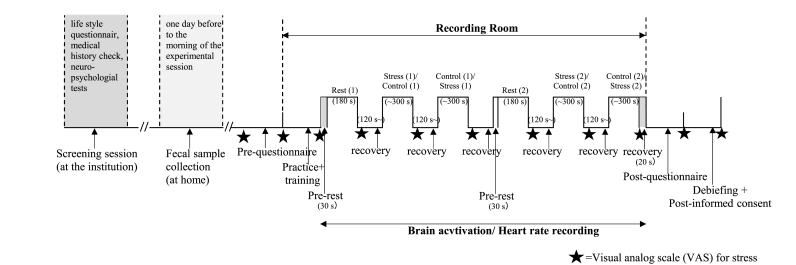
Timeline of the experimental session. The stars indicate the timepoints when the visual analog scale was presented.

**Fig. 2:**
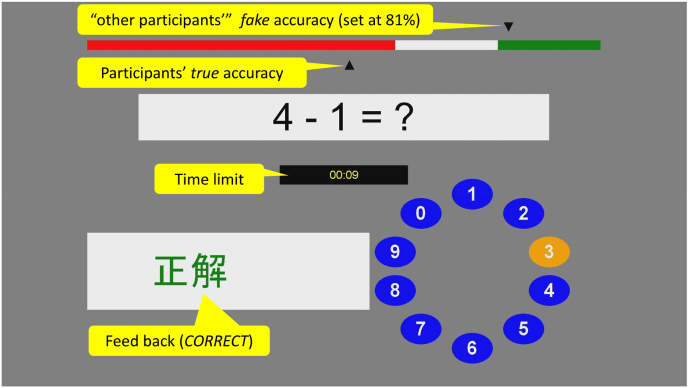
The graphical interface of the stress condition in the Montreal Imaging Stress Task. The figure shows the performance indicator, arithmetic task, time limit, feedback, and rotary dial for response submission.

Participants were subjected to psychosocial stress using the MIST program (Dedovic et al., 2005). The program was divided into practice, training, and experimental sessions (Fig. 1; Supplemental Material 3). The experimental session was divided into two parts, each consisting of a 3-min rest, 5-min control, and 5-min stress condition. In the rest condition, participants were required to fixate on the answering display that was presented. In the control and stress conditions, participants were instructed to answer arithmetic tasks which consisted of five difficulty levels (approximately 1-min each) in random order. Subsequent questions were presented after the feedback (CORRECT, ERROR or TIMEOUT!). In the control condition, participants answered the arithmetic questions at their own pace. In the stress condition (Fig. 2), participants were informed that, for their scores to be accepted as valid data, their performance had to be equal to or higher than the average accuracy (81%). However, unbeknown to participants, time limits were dynamically set to ensure below average accuracy. To induce additional psychosocial stress, participants were shown both time limits and their accuracy, and negative comments were provided by the experimenter. After each condition, a visual analogue scale (VAS) was displayed with the question, “How stressed do you feel?“, and participants used the cursor to indicate their subjective stress level for the previous task.

After completing the MIST, participants completed the post-experiment questionnaires, and they were debriefed about the task. We obtained written informed consent to use the collected data. We calculated the mean accuracy and mean reaction time for the behavioral results and conducted a two-factor analysis of variance (ANOVA) with repetition of the experimental condition (control, stress) × task difficulty (1–5).

### 2.3 Psychological assessment

Before and after the MIST, the participants’ subjective stress during the task was measured using following questionnaires: Positive and Negative Affect Schedule (PANAS) (Watson et al., 1988), State Self-esteem Scale (SSES) (Heatherton and Polivy, 1991), Profile of Mood States 2 short version (POMS2), and the state anxiety questions of the State-Trait Anxiety Inventory (STAI) (Spielberger et al., 1983). Pre- and post-MIST scores were compared using paired *t*-tests. Participants were also asked to rate their subjective stress via the VAS at eight different timepoints pre- and post-MIST. For the analysis, VAS scores for the three experimental conditions were averaged by the first and second halves. These scores were used for repeated one-factor ANOVA.

### 2.4 Heart rate measurement and analysis

During the MIST, participants’ heart rate data were recorded with an electrocardiogram using a biosignal recording system (Polymate V AP-5148; Miyuki Giken, Co. Ltd., Tokyo, Japan). The sampling rate was 1000 Hz. The acquired heart rate data were analyzed using an R-R interval analysis program (R-R Interval, CDM Analysis; NoruPro Light systems, Inc., Tokyo, Japan). The low-cut filter for preprocessing was 3.00 Hz. We conducted one-factor ANOVA with repetition for beat per minute (bpm) during the rest, control, and stress conditions.

### 2.5 Brain activation data: acquisition and analysis

#### 2.5.1 fNIRS measurement

Hemodynamic responses were measured with the continuous wave multi-channel fNIRS system (OT-R40 Optical Topography System; Hitachi Medical Co., Tokyo, Japan) with a temporal resolution of 10 Hz. The device estimates changes in the hemoglobin (Hb) concentration and oxygenation levels of the optical paths in the underlying cortical areas between the nearest pairs of emitter and detector probes. Particularly, the instrument measured changes in oxy- and deoxygenated hemoglobin (deoxy-Hb) concentrations using continuous near-infrared lasers with the wavelength (approximately 695 nm and 830 nm, respectively) based on the Modified Beer-Lambert law. The relative changes in hemoglobin concentration from baseline to the activation period were indicated as mM·mm. We used oxy-Hb as an indicator of cortical activation because oxy-Hb better reflects cortical activity and has a stronger correlation with fMRI blood oxygenation level-dependent signals compared to deoxy-Hb (Strangman et al., 2002).

**Fig. 3:**
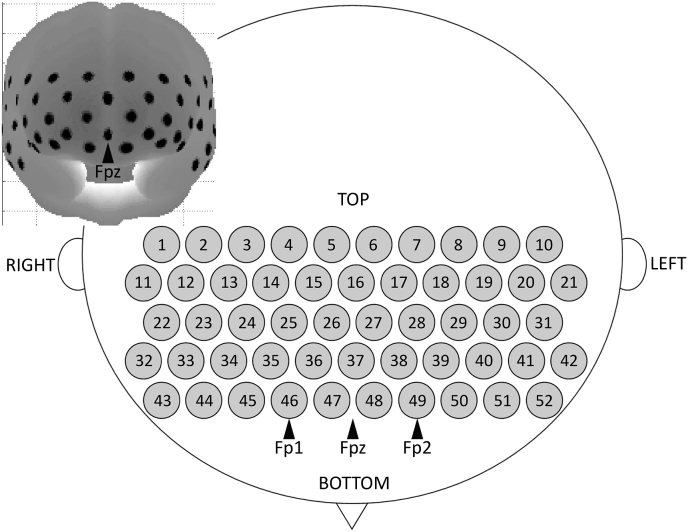
Probe setting and measurement points for the 52-channel functional near-infrared spectroscopy.

To measure cortical activation in the frontal and partial parietal areas, we used a thermos plastic probe (3 × 11 shell set) with 52 channels (CH) (Fig. 3). The distance between the source and the detector-pair was set at 30 mm. The lowest probe line was set along the Fp1-Fp2 line (as defined by the international 10–20 system used in electroencephalography), and the center of the CHs was positioned across the nasion-inion line. This probe arrangement enabled the spatial estimation of localized cerebral activity, based on the virtual registration method (Tsuzuki et al., 2007). After attaching the probe, the experimenter verified that each probe was properly placed and in contact with the scalp. The fNIRS recording was initiated, and measurements were obtained with the participants sitting in a chair with their eyes open and their head resting on the chinrest.

#### 2.5.2 fNIRS data analysis

The fNIRS data were preprocessed using the Platform for Optical Topography Analysis Tools (POTATo) software developed by the Research and Development Group of Hitach, Ltd. (Tokyo, Japan) in a MATLAB 2012a environment (MathWorks, Inc., Natick, MA, USA) I wanted to add a reference for MATLAB as mentioned in Q3. However, I couldn't successfully add it via free text (message asks for "own label" which I coudn't understand...). Here is the information of the program for the Reference: "MATLAB, Image Processing Toolbox, Signal Processing Toolbox and Statistics and Machine Learning Toolbox Release 2012a, The MathWorks, Inc., Natick, Massachusetts, United States." I'd really appreciate it if you can put this in appropriate form and add it to the Reference list. For each participant, the raw oxy-Hb data in each CH were high-pass filtered (cut-off frequency, 0.01 Hz) to remove baseline drift, and low-pass filtered (cut-off frequency, 0.5 Hz) to remove heartbeat pulsation.

For the rest condition, we conducted a block analysis on the 190-s epoch composed of a 10-s baseline period immediately before each condition block period and the 180-s target period of each condition block. For the stress and control conditions, the block analysis was focused on the 327-s epoch composed of the 10-s baseline period immediately before each condition block period and the 317-s target period of each condition block. The stress and control conditions were both originally set to 300-s per block, although the actual time differed between participants because of their response times. All participants had completed the task within 317-s, and thus the target periods were set to 317-s. The time-series of concentration changes was subtracted from the mean change during the 10-s baseline period. Epochs with motion artifacts and any blocks containing >30 s of continuous recording failure were excluded. The time-series of oxy-Hb concentration changes were averaged over epochs for each condition, CH, and participant. As a characteristic of the hemodynamic response, the first 5 s of all three experimental condition blocks were discarded from the analysis. We conducted paired *t*-tests for the changes in oxy-Hb concentrations [(the stress condition minus the rest condition) versus (the control condition minus the rest condition)] to identify the activated regions related to the psychosocial stress. We also conducted false discovery rate correction to correct for multiple comparisons among the 52 CHs. The brain regions underlying each CH were estimated using the virtual registration method for the fNIRS CHs (Tsuzuki et al., 2007). For the CHs that showed significant psychosocial stress-related activation, we analyzed the relationship between the microbiota at phylum and genus levels using multivariate analysis, adjusted for age and body mass index (BMI). We used an alpha level of .05 for all statistical tests, unless otherwise stated.

### 2.6 Fecal microbiota analysis

On the day before, or in the morning of the experimental session, fecal samples were collected from the participants in fecal sampling tubes containing a preservation solution. Samples were refrigerated for a maximum of 30 days and sent to Techno Suruga Laboratory Co., Ltd. (Shizuoka City, Japan) for analysis of the participants’ intestinal microbiota.

Deoxynucleic acid (DNA) was extracted, using a previously described method (Takahashi et al., 2014) and an automated DNA isolation system (GENE PREP STAR PI-480, Kurabo Industries, Ltd., Osaka, Japan). The V3–V4 regions of bacterial and archaeal 16S rRNA were amplified using the Pro341F/Pro805R primers and the dual-index method (Hisada et al., 2015; Takahashi et al., 2014). Barcoded amplicons were paired-end sequenced on a 2 × 284-bp cycle using the MiSeq system with MiSeq Reagent Kit v3 (600 cycles; Illumina, Inc., San Diego, CA, USA). Paired-end sequencing reads were merged using the Fastq-Join program with default settings (Aronesty, 2013).

Joined reads with a quality score of ≥20 for >99% of the sequences were extracted using the FASTX-Toolkit (Cambridge, UK). Chimeric sequences were deleted with USEARCH6.1 (Caporaso et al., 2010; Edgar et al., 2011). Analyses of sequence reads were conducted manually using the Ribosomal Database Project Multiclassifier tool Ver. 2.11 (http://rdp.cme.msu.edu/classifier/) (Wang et al., 2007). Bacterial and archaeal species were identified from the sequences by using the Metagenome@KIN Ver. 2.2.1 analysis software (World Fusion, Tokyo, Japan) and the TechnoSuruga Lab Microbial Identification database DB-BA Ver. 13.0 (TechnoSuruga Laboratory, Shizuoka City, Japan) with ≥97% homology (Kasai et al., 2015). The relationship between the microbial abundance at the phylum and genus levels with the psychosocial stress response were analyzed separately. We used an alpha level of .05 for all statistical tests.

### 2.7 Daily health log

The daily health log contained questions related to overall health status, dietary intake of prebiotics, probiotics and other fermented food, amount of exercise, and medication. Regarding dietary intake, participants were prohibited from taking any prebiotics, probiotics, or fermented food from five days before the experimental session.

## 3 Results

One participant experienced problems in attending the MIST sessions; therefore, the following analyses include data from a maximum of 59 participants. Demographic details are presented in Table 1.

**Table 1:** Demographic details and baseline characteristics of all participants.

	Min-Max	Mean	S.D.
Age	25–45	35.07	6.24
BMI	17.5–26.5	22.60	8.10
POMS	−20-48	8.12	16.22
BDI	0–12	2.93	3.26
Rosenberg Self-esteem Scale	22–50	37.46	6.56
Pittsburgh Sleep Questionnaire	0–9	3.15	1.98
Stress Response Scale	0–29	6.63	7.46
Perceived Stress Scale	0–41	22.65	9.18
Japanese Burnout Scale-E	5–22	9.67	4.13
Japanese Burnout Scale-D	6–21	9.98	4.08
Japanese Burnout Scale-PA	8–29	19.04	5.57
Mental calculation*	42–50	48.72	1.57
		***N***	**%**
Education	Secondary	4	6.8
	Undergraduate	43	72.9
	Postgraduate	12	20.3
Exercise habits	Yes	30	50.8
	No	29	49.2
Abdominal pain/	Yes	0	0.0
Abnormal bowel habits**	No	59	100.0

BDI, Beck Depression Inventory; BMI, body mass index; Japanese Bounout Scale-D, depersonalization; -E, emotional exhaustion; -PA, personal accomplishment; Min, minimum; Max, maximum; POMS, Profile of Mood States 2 short version; S.D., standard deviation.

* Participants solved the elementary school level (1–2 digits) addition, subtraction, multiplication, or division by mental arithmetic. Maximum score was 50.

** More than 3 consecutive days of abdominal pain/abnormal bowel habits within the past 3 months.

### 3.1 Task performance (accuracy and response time)

**Fig. 4:**
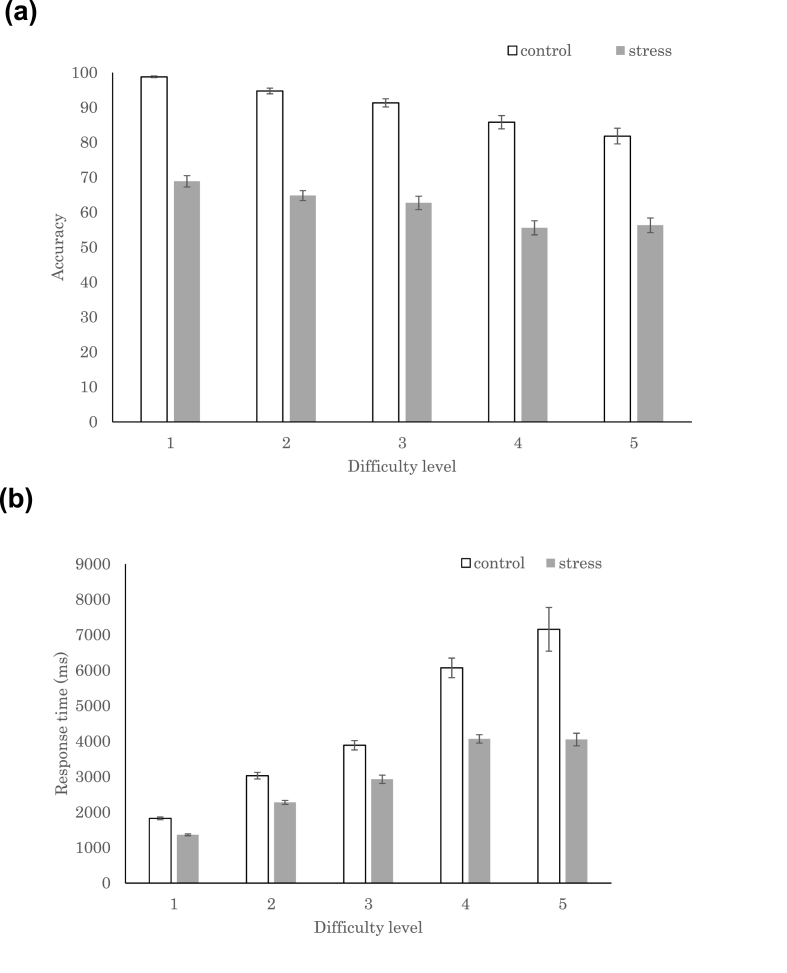
Performance of Montreal Imaging Stress Task in the control and stress conditions. **(A)** The accuracy and **(B)** the response times for the five difficulty levels. The error bars indicate 1 standard error.

We conducted a 2 × 5 two-way repeated ANOVA (experimental conditions: stress, control) × (difficulty levels: 1–5) to identify the effect of psychosocial stress and arithmetic difficulty levels on behavioral performance. We found that accuracy (experimental condition: *F*_(1,522)_ = 836.1, *p* < .0001; difficulty level: *F*_(4,522)_ = 30.8, *p* < .0001) and response time (experimental condition: *F*_(1,522)_ = 119.6, *p* < .0001; difficulty level: *F*_(4,522)_ = 125.2, *p* < .0001) were affected by psychosocial stress and difficulty levels (Fig. 4). Performance tended to be more accurate and response time was shorter when less stress existed and the arithmetical task was easier. The post-hoc Tukey analysis revealed that the mean accuracy was higher in the control than the stress condition, whereas the mean response time was longer in the control than the stress condition. The shorter response time in the stress condition was caused by the time limitation, which was absent in the control condition. Accuracy was higher for level 1 tasks than for levels 3, 4, and 5. Accuracy was higher for levels 2 and 3 than levels 4 and 5. Accuracy did not exhibit any interaction effects. Response times were significantly different between all pairwise comparisons, except for that of levels 4 and 5, which indicated a relatively shorter response time for the easier levels. Response time also had a significant interaction effect and was shorter for the stress condition than for the control condition in levels 3, 4, and 5.

### 3.2 Psychological assessment

#### 3.2.1 PANAS, POMS2, SSES, and STAI

Paired *t*-tests were conducted to compare the subjective stress levels before and after the MIST (Table 2). As expected, the PANAS results post-MIST indicated a decrease in positive emotion and an increase in negative emotion (positive emotion: *t*_(58)_ = -4.05, *p* < .001; negative emotion: *t*_(58)_ = 5.83, *p* < .0001). The post-MIST POMS2 results indicated increased total mood disturbance (*t*_(58)_ = 5.27, *p* < .0001), including increased depression (*t*_(58)_ = 4.00, *p* < .0001). The SSES results indicated decreased self-esteem (*t*_(58)_ = -3.97, *p* < .001), and the STAI results indicated increased anxiety (*t*_(58)_ = 4.60, *p* < .0001).

**Table 2:** Raw scores for the pre- and post-Montreal Imaging Stress Task responses on the questionnaires. Data are expressed as the mean (standard deviation). The *p* values are shown for each pairwise comparison.

Questionnaire:	Pre-MIST		Post-MIST		p-value
subscales					
**PANAS**					
Positive affect	30.71	(7.2)	26.14	(7.7)	<.001
Negative affect	19.36	(7.2)	23.95	(9.2)	<.001
**POMS2**					
anger	43.14	(7.6)	45.08	(8.8)	.083
confusion	45.25	(7.9)	51.17	(10.4)	<.0001
depression	46.00	(7.5)	50.17	(8.9)	<.001
fatigue	41.53	(6.9)	49.51	(9.6)	<.0001
tension	44.93	(8.8)	48.10	(9.2)	.0081
friendliness	54.12	811.2)	49.51	(12.2)	<.0001
vigor	53.27	(10.5)	48.39	(9.8)	<.0001
TMD	43.22	(8.2)	49.32	(9.7)	
**SSES**	67.83	(9.0)	63.54	(12.1)	<.001
**STAI**	41.34	(8.6)	47.41	(10.7)	<.0001

MIST, Montreal Imaging Stress Task; PANAS, Positive and Negative Affect Schedule.

POMS2, Profile of Mood States 2 short version; SSES, State Self-Esteem Scale.

STAI, State-Trait Anxiety Inventory; TMD, Total Mood Disturbance.

#### 3.2.2 VAS for subjective stress

**Fig. 5:**
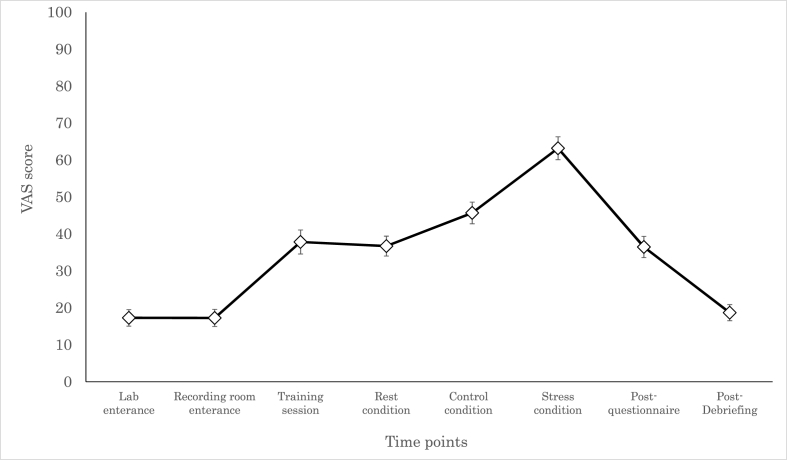
Trajectory of subjective stress responses throughout the experiment. The error bars indicate 1 standard error.

Participants answered the VAS question at eight different timepoints (Fig. 5). One-way ANOVA revealed that the participants’ perceived stress levels changed throughout the study (*F*_(7,405)_ = 75.6, *p* < .0001). The post-hoc Tukey analysis revealed that stress level was highest in stress conditions (*p* < .00001). Compared to the other timepoints, stress levels were higher for the stress and control conditions at the following timepoints: entering the room, entering the recording room, after the training, after the psychological assessment, and post-debriefing (all *p* < .05). In the rest condition, stress scores were higher for all timepoints, except after training.

### 3.3 Heart rate

**Fig. 6:**
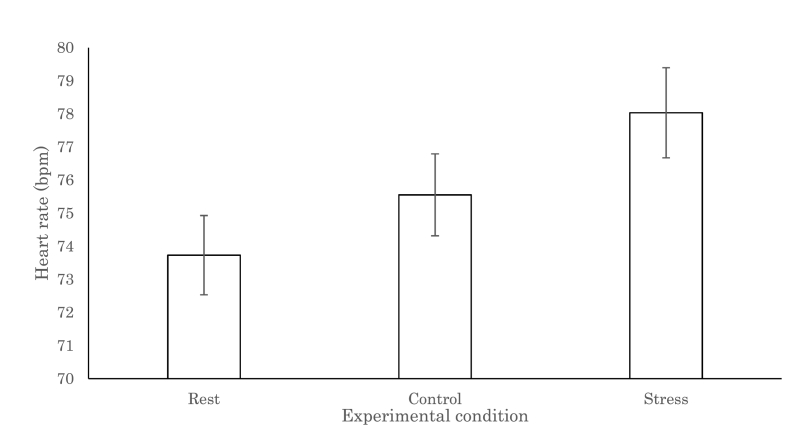
Changes in the heart rate per minute throughout the experiment. The error bars indicate 1 standard error.

The heart rate data of one participant were discarded because of recording failure. The following analysis was conducted using the data of 58 participants (Fig. 6). One-way ANOVA was conducted to evaluate the effect of psychosocial stress on bpm, revealing that heart rates differed among the three experimental conditions (*F*_(2,114)_ = 39.0, *p* < .0001). The post-hoc Tukey analysis revealed that heart rates were significantly higher in the stress condition than in the control and rest conditions (both, *p* < .0001). The difference between the control and rest conditions was also significant (*p* < .001).

### 3.4 Neural correlates of psychosocial stress: Stress–Rest versus Control–Rest contrasts

**Fig. 7:**
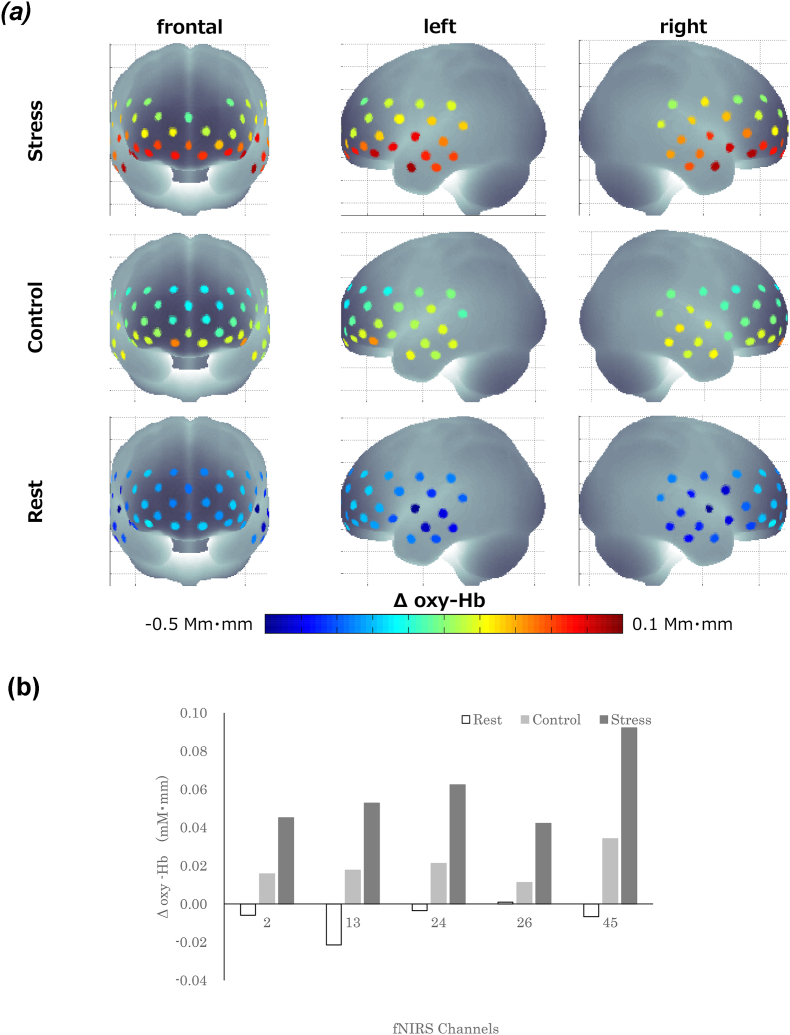
Brain activation during the three experimental conditions. **A**. Activation in the whole brain (hot colors indicate an increase in oxygenated hemoglobin [oxy-Hb] changes; cold colors indicate a decrease in oxy-Hb changes). **B**. Activation in the five functional near-infrared spectroscopy channels that showed stress-related brain activation. The error bars indicate 1 standard error. (For interpretation of the references to color in this figure legend, the reader is referred to the Web version of this article.)

To examine the neural correlates of psychosocial stress, we identified the activated brain regions by using paired *t*-tests for the mean oxy-Hb change: (the stress condition minus the rest condition) versus (the control condition minus the rest condition). Psychosocial stress-induced changes in mean oxy-Hb were observed in five CHs, all converging in the right hemisphere (Fig. 7). These areas included the right PMC/SMA (pre-motor cortex/supplementary motor area) (CH 2: *t*_(35)_ = -3.12, *p* < .05), the right dlPFC (dorsolateral prefrontal cortex) (CH 13: *t*_(33)_ = -3.42, *p* < .05; CH 24: *t*_(48)_ = -3.05, *p* < .05), the right FP (frontal pole) (CH 26: *t*_(45)_ = -3.21, *p* < .05), and the right IFG (inferior prefrontal gyrus) (CH 45: *t*_(28)_ = -3.20, *p* < .05). The mean oxy-Hb changes in all CHs were greater in the Stress–Rest contrast than in the Control–Rest contrast.

### 3.5 Relationship between the stress response and gut microbiota

**Fig. 8:**
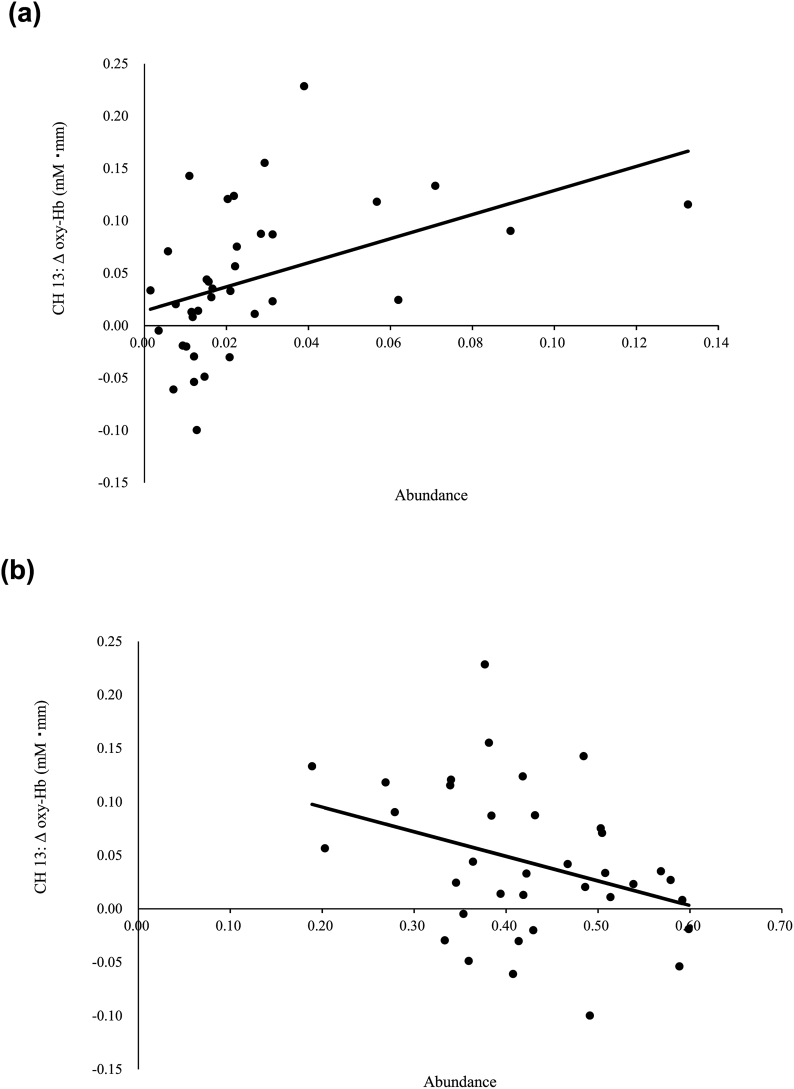
Correlation between the oxyhemoglobin [oxy-Hb] changes in channel 13 and **(A)** the abundance of *Proteobacteria* and **(B)** the abundance of *Firmicutes*.

One participant failed to submit a fecal sample. Thus, the following data were derived from 58 fecal samples for the analysis of gut microbiota. We analyzed the relationship between microbiota abundance at the phylum and genus levels by using multivariate analysis, controlled for age and BMI (Table 3). At the phylum level, the abundances of *Proteobacteria* and *Firmicutes* were associated with the oxy-Hb changes in CH 13, suggesting that participants with high psychosocial responses had a high abundance of *Proteobacteria* (β = 0.44; *p* = .013) (Fig. 8A) and low abundance of *Firmicutes* (*β* = −0.34; *p* = .043) (Fig. 8B). No other associations were observed for the phylum-level analysis (all, *p* > .05).

**Table 3:** Multivariate analysis of the association between oxygenated hemoglobin [oxy-Hb] changes in psychosocial stress-related brain areas and the gut microbiota, adjusted for age and body mass index.

	CH 2						CH 13						CH 24						CH 26						CH 45					
	*β*		95% Cl		p value	Adjusted R2	*β*		95% Cl		p value	Adjusted R2	*β*		95% Cl		p value	Adjusted R2	*β*		95% Cl		p value	Adjusted R2	*β*		95% Cl		p value	Adjusted R2
***Phyllum***																														
*Firmicutes*							−0.34	−0.452	-	−0.007	0.043	0.083																		
*Proteobacteria*							0.44	0.264	-	2.042	0.013	0.142																		
***GENUS***																														
*Acidaminococcus*																			0.39	1.487	-	10.207	0.010	0.128						
*Acinetobacter*	−0.34	−8414.294	-	−253.567	0.038	0.170																								
*Actinomyces*																			−0.29	−182.121	-	−0.088	0.050	0.068						
Alistipes*																			0.34	0.565	-	6.518	0.021	0.100	0.46	1.393	-	8.969	0.009	0.334
*Allisonella*	−0.39	−69.703	-	−8.195	0.015	0.213	−0.38	−76.196	-	−5.252	0.026	0.108	−0.29	−82.864	-	−3.888	0.032	0.194												
*Alloscardovia*	0.42	430.351	-	3645.521	0.015	0.213																								
*Anaerofilum*													0.31	89.713	-	954.292	0.019	0.210	0.36	139.886	-	1100.225	0.013	0.119	0.51	352.998	-	1321.832	0.002	0.415
*Asaccharobacter*																			0.41	267.103	-	1360.971	0.005	0.156						
Blautia*																									−0.49	−1.981	-	−0.373	0.006	0.356
Clostridium IV*																									0.48	1.830	-	9.739	0.006	0.355
Clostridium XI*																			−0.47	−7.812	-	−1.748	0.003	0.174						
*Corynebacterium*	0.47	266.807	-	1443.010	0.006	0.254	0.46	199.861	-	1523.101	0.012	0.144													0.39	97.177	-	2153.268	0.033	0.273
*Eisenbergiella*													0.35	19.617	-	130.247	0.009	0.232							0.37	10.465	-	167.426	0.028	
*Enterococcu*																									0.39	13.717	-	142.614	0.019	0.299
*Enterorhabdu*																			0.46	31.080	-	120.064	0.001	0.120						
Faecalibacterium*													−0.28	−1.574	-	−0.064	0.034	0.192												
*Hydrogenoanaerobacterium*																			0.33	231.541	-	3734.994	0.027	0.090						
*Methanomassiliicoccus*																									0.47	55.394	-	274.223	0.005	0.365
*Negativicoccus*	0.42	2582.119	-	21873.217	0.015	0.213	0.47	4446.212	-	28691.928	0.009	0.159																		
*Odoribacter*													0.28	0.425	-	29.681	0.044	0.184												
*Oligosphaera*													0.35	189.463	-	1127.402	0.007	0.240	0.38	165.582	-	1051.337	0.008	0.134	0.36	42.582	-	1009.605	0.034	0.271
*Oxalobacter*													0.28	26.947	-	770.489	0.036	0.190	0.40	129.056	-	740.005	0.006	0.143						
*Pantoea*	−0.36	−6326.126	-	−354.826	0.030	0.182																								
*Parasutterella*	−0.45	−17.219	-	−2.913	0.007	0.243																								
*Parvibacter*													0.35	521.021	-	3100.348	0.007	0.240	0.38	455.350	-	2891.171	0.008	0.134	0.36	117.101	-	2776.408	0.034	0.271
*Pasteurella*																			0.52	1244.083	-	3876.892	0.000							
*Pediococcus*	−0.35	−584.698	-	−16.866	0.039	0.170																								
*Peptococcus*																			0.52	44.963	-	139.315	0.000	0.248						
*Rejected hit*																									0.35	0.025	-	0.900	0.039	0.265
*Rikenella*													0.35	2084.087	-	12401.412	0.007	0.240	0.38	1821.402	-	11564.703	0.008	0.134	0.36	468.404	-	11105.647	0.034	
*Saccharibacteria_genera_incertae_sedis*	−0.36	−278.676	-	−11.505	0.034	0.175																								
*Saccharofermentans*																			0.31	265.572	-	7909.627	0.037	0.079	0.35	248.125	-	8407.943	0.039	0.271
*Shuttleworthia*																			0.32	1289.364	-	31641.795	0.034	0.082						
*Sphingomonas*													0.40	2636.166	-	10704.974	0.002	0.281	0.42	2082.244	-	9807.848	0.003	0.166	0.35	267.602	-	8881.770	0.038	0.266
*Succinatimonas*																			0.42	14.115	-	77.869	0.006	0.147						
Sutterella*	0.33	0.097	-	4.181	0.041	0.167																								
*Treponema*													0.35	12.391	-	73.439	0.007	0.240	0.38	10.742	-	68.413	0.008	0.134	0.36	2.873	-	65.806	0.034	0.272
*Varibaculum*	−0.36	−4599.928	-	−298.208	0.027	0.186																								
*Veillonella*	−0.36	−3.195	-	−0.223	0.026	0.188																								
*Victivallis*																			0.29	2.500	-	474.043	0.048	0.069

At the genus level, the abundances of 40 genera were associated with the oxy-Hb changes in psychosocial stress-related brain areas. These genera included six predominant genera (defined in this study as ≥0.5% of the total sequences) and 34 less predominant genera (Table 3). Among the predominant genera the oxy-Hb changes were associated with *Sutterella* (*β* = 0.33; *p* = .041) in CH 2 (right PMC/SMA); *Faecalibacterium* (*β* = −0.28; *p* = .034) in CH 24 (right dlPFC); *Alistipes* (*β* = 0.34; *p* = .021) and *Clostridium XI* (*β* = −0.47; *p* = .003) in CH 26 (right FP); and *Clostridium IV* (*β=*0.48; *p* = .006)*, Alistipes* (*β* = 0.46; *p* = .009), and *Blautia* (*β* = −0.49; *p* = .006) in CH 45 (right IFG). We also performed further analyses that were additionally controlled for participants’ dietary intake, exercise, and baseline mood; however, none of these factors had a significant effect (Supplemental Material 4).

## 4 Discussion

In this study, we investigated the relationship between psychosocial stress-related brain activation and gut microbiota in healthy participants, analyzing their behavioral data, physiological responses, and subjective stress during the MIST. As expected, psychological stress increased subjective stress and anxiety, and reduced self-esteem. We also observed decreased behavioral performance, increased heart rate, increased sympathetic activity in line with the subjective results, and enhanced activity in the right PMC/SMA, right dlPFC, right FP, and right IFG. Notably, changes in oxy-Hb concentration in psychosocial stress-activated brain regions were associated with the abundance of several gut microbes, indicating a robust relationship between psychosocial stress and gut microbiota composition in healthy participants.

### 4.1 Psychosocial stress response as brain activation in healthy participants

The MIST results showed that psychosocial stress was perceived as subjective stress and expressed through physiological responses. All activated brain regions associated with psychosocial stress converged in the right hemisphere and included the PMC/SMA, dlPFC, FP, and IFG. This is consistent with the results of previous studies (Elliot et al., 2002; Jankowski et al., 2018; Li et al., 2019) investigating the neural basis of psychosocial stress. The right PMC/SMA is associated with social rejection and anxiety (Jankowski et al., 2018; Li et al., 2019). The right dlPFC and right IFG are associated with emotional processing in psychosocial stress responses. Under stress, these areas are more active in MDD (major depressive disorder) than healthy participants (Elliot et al., 2002; Fitzgerald et al., 2006; Jankowski et al., 2018). Additionally, the right FP is associated with stress perception, increased heart rate, and increased cortisol secretion (Wang et al., 2005). The functions of these brain regions support the hypothesis that the changes in brain activity observed in this study were induced by psychosocial stress. Furthermore, HPA-axis activity is primarily controlled by the right PFC. Therefore, participants with higher psychosocial stress-induced activity in the right PFC also may have had relatively high HPA-axis activity (Cerqueira et al., 2008). As patients with depression often exhibit HPA-axis overactivation, we infer that participants with higher psychosocial stress-induced brain activation were generally more vulnerable to psychosocial stress and more likely to overreact to negative feedback.

### 4.2 Relationship between stress response and gut microbiota

At the phylum level, activation of the right dlPFC showed positive and negative associations with the abundances of *Proteobacteria* and *Firmicutes*, respectively. This finding was consistent with the results of a study on mice that had been subjected to psychosocial stress (Geng et al., 2020). Human studies also indicate that the abundance of *Proteobacteria* (Jiang et al., 2015) is higher and that of *Firmicutes* (Huang et al., 2018; Jiang et al., 2015) is lower in patients with depression. The right dlPFC is associated with psychosocial stress and social exclusion and is more active in MDD than healthy participants (Jankowski et al., 2018; Li et al., 2019). Thus, people with a high abundance of *Protobacteria* and/or low abundance of *Firmicutes* would likely have a high psychosocial stress response.

A similar analysis using genus-level classifications also revealed an association between psychosocial stress-induced brain activity and 40 genera. Notably, 17 of these genera are associated with stress, depression, and anxiety (Jiang et al., 2015; Kelly et al., 2016; Liu et al., 2016; Messaoudi et al., 2011). The abundance of *Alistipes*, *Anaerofilum*, *Asaccharobacter*, *Clostridium IV*, *Odoribacter*, and *Oxalobacter* were positively associated with activation in the right dlPFC and right FP. These genera are more prevalent in patients with depression or MDD than healthy individuals, and their abundance is positively correlated with anxiety and stress load (Barandouzi et al., 2020; Bendtsen et al., 2012; Huang et al., 2018; Jiang et al., 2015; Kelly et al., 2016). *Clostridium XI* and *Faecalibacterium* abundances were negatively associated with activations in these two brain regions. These microbes are less prevalent in patients with depression. Particularly, the abundance of *Faecalibacterium* is negatively correlated with depression symptoms, suggesting that individuals with low *Faecalibacterium* abundance are more vulnerable to psychosocial stress than individuals with high *Faecalibacterium* abundance (Huang et al., 2018; Jiang et al., 2015; Liu et al., 2016). The abundance of *Blautia* is negatively associated with activation in the right IFG, and *Blautia* microbes are more prevalent in MDD (Jiang et al., 2015). The right IFG suppresses and controls negative emotions; therefore, participants with high *Blautia* abundance may have a strong tendency for pessimistic rumination because of their inability to control negative emotions (Rosenbaum et al., 2018). Such pessimistic rumination can trigger stress (Du et al., 2018; Rosenbaum et al., 2018) and, if continued, may lead to the onset of depression and affect its severity (Michalak et al., 2011; Smith and Alloy, 2009). Taken together, these results suggest that a significant association exists between the abundance of microbial genera and brain responses to psychosocial stress in healthy, nondepressed participants.

We assume that the underlying mechanism of the above-mentioned association involves HPA-axis activity and CRH secretion, which are important factors in bidirectional pathway of the microbiota-gut-brain axis (Wu et al., 2021). Studies using mice models have reported that stress promotes colonic motility via the HPA-axis and alters gut microbiota composition (Park et al., 2013). The altered gut microbiota in high responding participants may have induced inflammation in the intestinal tract and affected blood tryptophan levels, serotonin levels in the PFC, and dopamine metabolites in the cortex, further enhancing participants’ vulnerability to psychosocial stress (Foster and McVey Neufeld, 2013; Winter et al., 2018; Wu et al., 2021). This increased inflammation in healthy participants may be comparable to the enhanced inflammatory cytokine production in patients with depression. It is inferred that the common mechanism underlying both high responding participants and patients with depression have low *Faecalibacterium* abundance, the microbiota that increases Short-chain fatty acids including butyrate which plays an important role in regulation of inflammatory responses and induce anti-inflammatory, anxiolytic, and antidepressant like effects (Alameddine et al., 2019; Hao et al., 2019). Further, *Faecalibacterium* is negatively correlated with depression severity (Jiang et al., 2015). Additionally, *Alistipes* and *Entercoccus,* which affect glutamate and Gamma Amino Butyric Acid (GABA) metabolism, may be associated with increases in GABA and glutamate related genes in the dlPFC in MDD. This disruption of GABAergic gene expression may be the probable cause of GABA deficiency in MDD (Zhao et al., 2018). Taken together with our results, we speculate that, even in healthy participants, stress can cause changes in gut microbiota and PFC functioning. More importantly, the above-mentioned mechanisms in high responding participants yielded gut microbiota genera similar to those in patients with depression.

Accordingly, *Faecalibacterium*, *Alistipes,* and *Entercoccus* may contribute to predicting the depression phenotypes and symptoms. Specifically, as an equivalent amount of brain GABA is observed in Bipolar disorder (BD) and healthy controls (Schur et al., 2016), investigating the balance of *Faecalibacterium, Alistipes,* and *Entercoccus* abundance may help discriminate MDD onset from BD (McGuinness et al., 2022). Additionally, several distinct neural patterns in the PFC reportedly exist between MDD and BD (Han et al., 2019). Therefore, the combination of the gut microbiota balance and brain response in the PFC may improve the prediction accuracy of these disorders. However, as the present study included only healthy participants, comparative analysis of brain activation and gut microbiota in different psychiatric disorders under same conditions are necessary. Microbiota which may be used to distinguish healthy individuals from patients with depression are discussed in the following sections.

*Corynebacterium*, *Enterococcus*, and *Sutterella* abundances were positively associated with activation in the right PMC/SMA, right dlPFC, and right IFG. They are less abundant in patients with depression and MDD (Liu et al., 2016). Contrastingly, *Acinetobacter*, *Actinomyces*, *Allisonella*, *Parasutterella*, and *Veillonella*, which were negatively associated with the aforementioned regions, are reportedly more abundant in patients with depression or MDD. These results seem to contradict initial expectations; however, these genera may be non-resilient types, explaining these results.

The resilience of gut microbiota allows an organism to cope with and recover from stress-induced changes and disruptions (Dogra et al., 2020). Gut microbiota that can recover to their pre-stressed, baseline state are called “resilient gut microbiota,” whereas those that change to a new state are called “non-resilient gut microbiota.” Assuming that our seemingly contradictory results were caused by non-resilient genera, a possibility is that the abundance of *Corynebacterium, Enterococcus*, and *Sutterella* is low when the stress response is moderate or low. When the stress response is increased, microbiota resilience minimizes the damage by rapidly increasing the *Corynebacterium, Enterococcus*, and *Sutterella*. However, if the organism cannot recover to the baseline state and develops depression after chronic stress, the increased abundance of these genera would drop rapidly below the baseline, resulting in a stable but disrupted state. Indeed, Zhang et al. (2019) reported that *Corynebacterium* abundance increased (although not significantly) from baseline in rodents with stress avoidance behavior, but decreased in rodents without stress avoidance behavior. The abundance of *Acinetobacter, Actinomyces, Allisonella, Parasutterella*, and *Veillonellz* was high when the stress response was moderate or low; therefore, the reverse phenomenon is expected. Their abundance decreases when the stress response is high and increases rapidly with the development of depression.

Finally, out of the 40 identified genera in this study, 23 have not previously been associated with stress, anxiety, or depression. However, two genera—*Pantoea* and *Pediococcus*—may elicit a positive effect on psychosocial stress. *Pantoea* spp. are negatively associated with psychosocial stress responses in the right PMC/SMA, and is already used for cancer treatment and reversing immunosuppression. Studies (Walterson and Stavrinides, 2015) in mice and chickens have shown that components derived from *Pantoea* promote immune-related functions. Chronic stress and depression are associated with dysfunction of the immune system; therefore, an increase in *Pantoea* may decrease the stress response or enhance stress resilience. *Pediococcus* is used in food fermentation and negatively associated with psychosocial stress responses in the right PMC/SMA. Tomato extracts fermented with the *Pediococcus pentosaceus* OS strain have been shown to improve constipation symptoms and increase the abundance of *Bifidobacterium*, which has beneficial effects on the intestinal flora (Yoshinaga et al., 2014). The abundance of *Bifidobacterium* is lower in patients with depression than healthy participants; therefore, this *Pediococcus* induced abundance of *Bifidobacterium*, may help decrease stress responses or enhance stress resilience in individuals with a high stress response. The effect of *Pantoea* and *Pediococcus* on the stress response in humans needs further examination. Nevertheless, these genera are worth considering as potential candidates for stress reduction and resilience enhancement.

Importantly, it is worth noting that oxy-Hb changes were found to be significantly associated with the microbiota abundance in several brain regions, albeit by relative modest effect sizes. This is likely due to the fact that brain activation is not only associated with a stress response but also other mental manipulations, such as threat relevance (CH 2) (Portugal et al., 2020), attentional operation (CH 13) (Yamasaki et al., 2002), emotional suppression (CH 13, 24) (Friese et al., 2013), evaluation of the implication of negative events for future consequences (CH 45) (Kringelbach and Rolls, 2004), computation of contextual relevance of emotional information for decision making (CH 45) (Beer et al., 2006), and other irrelevant noise caused by attachment of the probe and slight body movement during the measurement. Thus, the present findings indicate that the brain response to stress is associated with the microbiota, despite the involvement of various other factors, which would have important implications for future research.

### 4.3 Limitations

This study had some limitations. First, this study only included males to control for the gender variance effect on stress. However, because stress responses and gut microbiota composition differ by sex, conducting similar experiments on male and female individuals in the future is important (Allen et al., 2017). Second, participants were all young to middle-aged. Previous studies have indicated that the subjective perceptions of stress and gut microbiota composition differ by age (Odamaki et al., 2016). Therefore, conducting experiments with a wider range of age groups is also important. Finally, we used fNIRS to measure brain functional activity to minimize unnecessary stress. Therefore, we could not capture activity in the deeper parts of the brain (e.g., the amygdala and insula) that constitute a central region for stress and emotional responses.

## 5 Conclusions

In this study, we recorded the brain activity of healthy participants subjected to psychosocial stress and examined the relationship between stress-related brain functions and the gut microbiota. Results showed that the PMC/SMA, dlPFC, IFG, and FP of the right hemisphere were involved in psychosocial stress responses. Furthermore, healthy participants with higher stress responses showed a higher abundance of gut microbes known to be abundant in patients with depression. Suggesting that healthy participants who are vulnerable to stress may have intestinal conditions similar to those of patients with depression. The gut microbiota positively associated with psychosocial stress in this study may be utilized as markers for the prediction of stress-induced diseases. Moreover, gut microbiota negatively associated with psychosocial stress may be used to enhance individual resilience to psychosocial stress and mitigate the subsequent risk of developing depression and anxiety disorders. Although future studies elucidating on our results are essential, we believe that this study is the first step in defining the direct relationship between brain function and the gut microbiota in healthy participants.

## Funding

This research did not receive any specific grant from funding agencies in the public, commercial, or not-for-profit sectors.

## CRediT authorship contribution statement

**Kao Yamaoka:** Conceptualization, Methodology, Investigation, Data curation, Resources, Writing – original draft, Writing – review & editing, Visualization, Project administration. **Nobuo Uotsu:** Resources, Supervision. **Eiichi Hoshino:** Methodology, Software, Formal analysis, Data curation, Writing – review & editing.

## Declaration of competing interest

The authors declare that they have no known competing financial interests or personal relationships that could have appeared to influence the work reported in this paper.

## Data Availability

Data will be made available on request.
