# Supplementary material for: Relationship between psychosocial stress-induced prefrontal cortex activity and gut microbiota in healthy Participants—A functional near-infrared spectroscopy study
Source: Neurobiol Stress. 2022 Aug 12;20:100479. doi: 10.1016/j.ynstr.2022.100479 (PMC9418982; doi:10.1016/j.ynstr.2022.100479)
Supplement: Multimedia component 1 [file mmc1.docx]

**Supplemental material 1. List of neuropsychological tests and questionnaires used in the screening test.**

**Supplemental material 2. Inclusion and exclusion criteria for the participants:**

Inclusion criteria:

1. Males 25 to 45 years of age
2. Daytime workers
3. Right handed

Exclusion criteria:

1. Color-vision deficiency,
2. Deficient visual acuity.
3. Hearing impairment.
4. MMSE score lower than 25.
5. Symptoms of previous diagnosis of dementia.
6. Any kind of recorded cerebral nerve disorders.
7. BDI score higher than 13.
8. Having or had dysregulation problems related to hypothalamic-pituitary-adrenal axis.
9. Undergoing hormone- related treatment.
10. Having or had achlorhydria.
11. Undergone digestive system surgery.
12. Had both day and nightshift during the study period
13. Vegans or vegetarians.
14. Smokers, or those who quit smoking in the last 12 months.
15. Attended a stress or emotion related study in the past 12 months.
16. Undergoing brain related treatment or prescribed related medicines.
17. Aware of sleep-related diseases such as sleep apnea, restless legs syndrome, and insomnia.
18. Taking medicines, health foods, foods for specified health uses, foods with nutrient function claims, and foods with functional claims that contain ingredients that may affect the study results, once or more times a week.
19. Cannot stop taking any of the above food in from 5 days before the day of testing.
20. Regular diarrhea or constipation.
21. Do not defecate more than 3 times a week.
22. Took antibiotics within 2 months of beginning of the study.
23. Donated or transfused 200 mL, 400 mL, or whole blood within the last 3 months
24. Participated in other clinical studies within one month, or are planning to participate other clinical studies during the study period.
25. Have disease that requires constant medication, or those have a serious medical history that requires medication.
26. Are judged to be unsuitable participant based on the examination results.
27. Works, or family works for a functional food -related company.
28. Judged by the research institute to be inappropriate candidates.

**Supplemental material 3. Experimental details for the Montreal Imaging Stress Task.**

On entering the recording room, the participants were seated with their head on a chinrest at a distance of 80 cm from the display screen. First, the instructions for the three experimental conditions were displayed. In the practice session, the participants performed the rest condition, followed by the easiest level of the arithmetic task in the stress condition (1 min) and control (1 min) condition using the numbers 0–9 on a rotary dial (visual angle: ~18.46° × 1.43° [horizontal × vertical]) . Participants selected their answers with a mouse. Subsequent questions were presented after the feedback (500 ms; CORRECT, ERROR).

During the training session, the participants performed the arithmetic task. There were five different levels of difficulty in the arithmetic task from level 1 (easy) to 5 (difficult) (examples of level 1 = 7-2, level 2 = 3-3+4, level 3 = 48+3-43, level 4 = 37-37*1+1, level 5 = 84*99/84-94). Each difficulty level lasted for 36 s under the control condition. The original time limit of the stress condition in the experimental session was determined, based on 90% of the average response time of the training session. We then attached electrodes and fNIRS probes to the participants to record their heart rate and brain activation data, respectively. The participants were instructed to limit their head and body movements during the experiment. In the experimental session, the rest condition was always performed first, but the order of the stress and control conditions (approximately 5 minutes) were counterbalanced among the participants. The time limit for stress condition was set for each question, and the remaining time was indicated in the center of the display (Figure 2). When the participants failed to respond within the time limit, subsequent questions were presented after the feedback (500 ms; TIMEOUT!). In the stress condition, a graph indicating the accuracy of the participant’s responses was displayed above the arithmetic question to induce psychosocial stress (Figure 2). However, the aforementioned instructions and the average accuracy were fake and were controlled to induce psychosocial stress. After each condition, the VAS question was displayed after a recovery period (fixation; 20 s) in which participants rated their subjective stress for the previous condition. Another recovery period (80 s) then followed.

**Supplemental material 4. Analysis results controlled for participants’ dietary intake, exercise, and baseline mood.**

Additionally, to confirm the effect of dietary intake, exercise, and baseline mood of the participants on results, we compared the original model adjusted for age and BMI with 4 other models by likelihood ratio tests: 1) age, BMI, and dietary intake, 2) age, BMI, and exercise, 3) age, BMI, and POMS2 score at the screening session, and 4) age, BMI, dietary intake, exercise, and POMS2 score at the screening session. For both phylum and genus level, the results showed that models 1 – 4 were not significantly different from the original model (*p* <.05). Further important point to note is that the original model had lowest AICc values for almost all the analyses. Altogether, these results suggests that the dietary intake, exercise, and baseline mood of the participants had little or no effect on our present results, and that the original model is the best fitted model for the current analysis.

AICc, Akaike Information Criterion corrected for small samples

BMI, body mass index

fNIRS, functional near-infrared spectroscopy

POMS2, Profile of Mood States 2 short version

VAS, visual analogue scale

**References**

Beck, A.T., Ward, C.H., Mendelson, M., Mock, J., Erabaugh, J., 1961. An inventory for measuring depression. Arch. Gen. Psychiatry. 4, 561–571. https://doi.org/10.1001/archpsyc.1961.01710120031004.

Buysse, D.J., Reynolds 3rd, C.F., Monk, T.H., Berman, S.R., Kupfer, D.J., 1989. The Pittsburgh Sleep Quality Index: a new instrument for psychiatric practice and research. Psychiatry Res. 28, 193–213. https://doi.org/ 10.1016/0165-1781(89)90047-4.

Cohen, S., Kamarck, T.P., Mermelstein, R., 1983. A global measure of perceived stress. J. Health Soc. Behav. 24, 385–396.

Folstein, M.F., Folstein, S.E., McHugh, P.R., 1975. “Mini-mental state”. A practical method for grading the cognitive state of patients for the clinician. J. Psychiatr. Res. 12, 189–198. https://doi.org/10.1016/0022-3956(75)90026-6.

Heuchert, J.P., McNair, D.M., 2012. Profile of Mood States–Second Edition (POMS-2). Multi-Health Systems Inc., Toronto, ON, Canada.

Kubo, M., 2014. The factorial and construct validity of the Japanese burnout scale. Shinrigaku kenkyu: The Japanese journal of psychology. 85, 364-372. https://doi.org/10.4992/jjpsy.85.13214. In Japanese.Lacy, B.E., Mearin, F., Chang, L., Chey, W.D., Lembo, A.J., Simren, M., Spiller, R., 2016. Bowel disorders. Gastroenterology. 150, 1393–1407. https://doi.org/10.1053/j.gastro.2016.02.031.

Nicholls, M.E., Thomas, N.A., Loetscher, T., Grimshaw, G.M., 2013. The Flinders Handedness Survey (FLANDERS): a brief measure of skilled hand preference. Cortex. 49, 2914–2926. https://doi.org/10.1016/j.cortex.2013.02.002.

Okubo, M., Suzuki, H., Nicholls, M.E.R., 2014. A Japanese version of the FLANDERS handedness questionnaire. Shinrigaku kenkyu: The Japanese journal of psychology. 85, 474–481. https://doi.org/10.4992/jjpsy.85.13235. In Japanese.

Rosenberg, M., 1965. Society and the Adolescent Self-image. University Press, Princeton, NJ, USA.

Suzuki, A., Shimada, H., Miura, M., Katayanagi, K., Umano, R., Sakano, Y., 1997. Development of a new psychological stress response scale (SRS-18) and investigation of the reliability and the validity. Jpn. J. Behav. Med. 4, 22–29. https://doi.org/ 10.4103/2347-5625.204494.
